# Bacteria tracking by *in vivo* magnetic resonance imaging

**DOI:** 10.1186/1741-7007-11-63

**Published:** 2013-05-28

**Authors:** Verena Hoerr, Lorena Tuchscherr, Jana Hüve, Nadine Nippe, Karin Loser, Nataliya Glyvuk, Yaroslav Tsytsyura, Michael Holtkamp, Cord Sunderkötter, Uwe Karst, Jürgen Klingauf, Georg Peters, Bettina Löffler, Cornelius Faber

**Affiliations:** 1 Department of Clinical Radiology, University Hospital Münster, Münster 48149, Germany; 2 Institute of Medical Microbiology, University Hospital Münster, Münster 48149, Germany; 3 Fluorescence Microscopy Facility Münster, Institute of Medical Physics and Biophysics, University Hospital Münster, Münster 48149, Germany; 4 Department of Dermatology, University Hospital Münster, Münster 48149, Germany; 5 Institute of Medical Physics and Biophysics, University Hospital Münster, Münster 48149, Germany; 6 Institute of Inorganic and Analytical Chemistry, University of Münster, Münster 48149, Germany

**Keywords:** Bacterial cell labeling, Bacteria tracking, Infectious diseases, Mouse models of infection, Cellular and molecular MRI

## Abstract

**Background:**

Different non-invasive real-time imaging techniques have been developed over the last decades to study bacterial pathogenic mechanisms in mouse models by following infections over a time course. *In vivo* investigations of bacterial infections previously relied mostly on bioluminescence imaging (BLI), which is able to localize metabolically active bacteria, but provides no data on the status of the involved organs in the infected host organism. In this study we established an *in vivo* imaging platform by magnetic resonance imaging (MRI) for tracking bacteria in mouse models of infection to study infection biology of clinically relevant bacteria.

**Results:**

We have developed a method to label Gram-positive and Gram-negative bacteria with iron oxide nano particles and detected and pursued these with MRI. The key step for successful labeling was to manipulate the bacterial surface charge by producing electro-competent cells enabling charge interactions between the iron particles and the cell wall. Different particle sizes and coatings were tested for their ability to attach to the cell wall and possible labeling mechanisms were elaborated by comparing Gram-positive and -negative bacterial characteristics. With 5-nm citrate-coated particles an iron load of 0.015 ± 0.002 pg Fe/bacterial cell was achieved for *Staphylococcus aureus*. In both a subcutaneous and a systemic infection model induced by iron-labeled *S. aureus* bacteria, high resolution MR images allowed for bacterial tracking and provided information on the morphology of organs and the inflammatory response.

**Conclusion:**

Labeled with iron oxide particles, *in vivo* detection of small *S. aureus* colonies in infection models is feasible by MRI and provides a versatile tool to follow bacterial infections *in vivo*. The established cell labeling strategy can easily be transferred to other bacterial species and thus provides a conceptual advance in the field of molecular MRI.

## Background

Bacterial infections remain one of the major challenges to human societies around the world. In particular, the emergence of multiple antibiotic resistant bacteria, which are difficult to treat and which can spread locally and systemically in different organs within the infected host, requires novel approaches for rapid diagnosis and efficient treatment [1-3]. Also, a deeper understanding of pathogen biology as well as of the complex host-pathogen interactions between bacteria and host tissues are urgently required to improve antimicrobial strategies. *Staphylococcus aureus* is a clinically highly relevant human pathogen, which has the potential to infect almost every organ leading to a number of diverse infections [4], such as skin and wound infections, pneumonia, endocarditis and osteomyelitis [5,6]. *S. aureus* induces a strong immune response either via a plethora of toxins and superantigens [7], complement activation, or by activation of the inflammasome and the related sterile injury danger signal pathways. Yet, the dynamics of dissemination and the exact bacterial strategies to establish metastasizing infections in different organs are largely unknown.

For research on dissemination dynamics in the entire host organism, murine models of bacterial infections are pivotal. Non-invasive *in vivo* imaging techniques, such as magnetic resonance imaging (MRI), computed tomography (CT), fluorescence/bioluminescence imaging (BLI) or positron emission tomography (PET), have been employed to visualize infections over the time course [8-12]. Although different strategies for specific detection of bacteria have been developed [13-15], most imaging studies target host factors [16] or detect immune cells [17]. Due to its high spatial resolution and excellent soft tissue contrast, especially MRI is a versatile method to image inflammatory processes upon bacterial infections non-invasively. While native MRI provides diagnostic information on tissue structures and the inflammatory status [18], detection of distinct cellular populations requires labeling with a suitable contrast agent, such as iron oxide particles [19,20]. Cell labeling and tracking experiments are commonly performed either by labeling immune cells *in vitro* and regrafting them or by systemic application of iron oxide particles which are then phagocytosed by macrophages [21]. By using negative iron contrast immunological reactions and bacteria-induced inflammatory dissemination have already been investigated in animal models of *Staphylococcus aureus*[22-26], *Toxoplasma gondii*[27], *Mycobacterium tuberculosis*[28] or LPS-induced sepsis conditions [29,30]. However, only BLI so far was capable of visualizing bacteria directly. This technique exploits detection of light emitted from luminescent proteins in transgenic and metabolically active bacteria. Owing to this unique property, BLI has become the most important and wide spread modality of detecting bacteria *in vivo*[31]. Here, we propose a complementary imaging modality based on MRI that requires the application of pre-treated bacteria, but which is also suitable for metabolically inactive or anaerobic pathogens and which provides detailed information about the bacterial localization in tissue. While native MRI provides diagnostic information on tissue structures and the inflammatory status, detection of distinct cellular populations requires labeling with a suitable contrast agent. We have used bimodal iron oxide nano particles (IONPs) to detect bacteria and have monitored dissemination in mouse models of local and systemic *S. aureus* infection. While for eukaryotic cells a variety of labeling protocols for cell imaging have been established, bacteria require specific labeling procedures that account for the presence of the bacterial cell wall. We have implemented a protocol that affords stable labeling of *S. aureus* cells with rhodamine-labeled 5-nm citrate-coated IONPs.

## Results and discussion

### Labeling strategy

**Figure 1:**
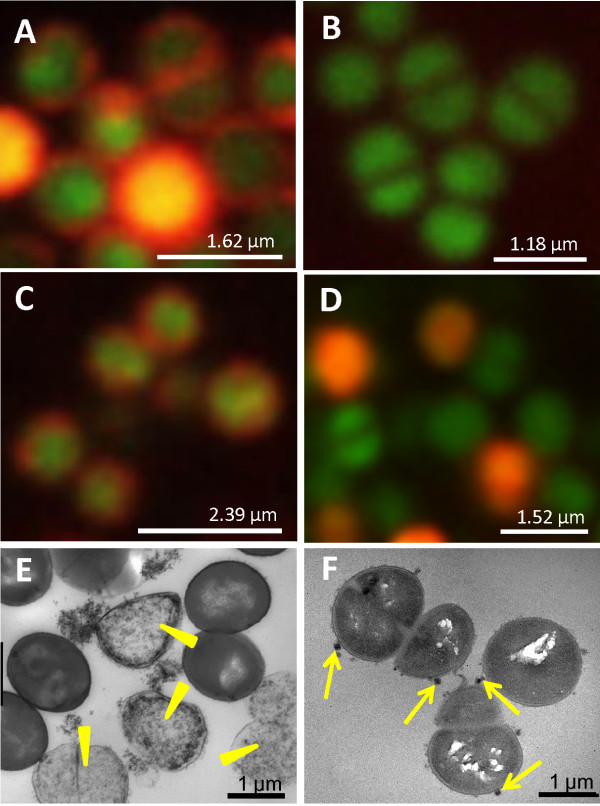
**Validation of bacterial cell labeling with iron oxide particles.** Fluorescence microscopy of *S. aureus* bacteria (strain 6850) labeled with Syto 9 (green) and rhodamine-labeled 5-nm citrate-coated iron oxide nano particles (IONPs) (red) using different labeling strategies: (**A**) electroporation of electro-competent bacterial cells, (**B**) incubation of unmodified bacterial cells and (**C**) incubation of electro-competent bacterial cells. Syto 9 was used for co-registration. (**D**) *S. aureus* bacteria labeled with 5-nm amine-coated IONPs by incubation of electro-competent bacterial cells. Electron microscopy images of *S. aureus* bacterial cell samples labeled by (**E**) electroporation and (**F**) incubation show a high percentage of cell debris when the electroporation technique was used (yellow arrowheads in (**E**)) and clusters of IONPs bound to the surface of intact bacteria (yellow arrows in (**F**)).

Depending on the phagocytotic cell characteristics, eukaryotic cells are commonly labeled either by incubation, transfection or electroporation [21]. To establish a labeling protocol for bacteria with MRI-sensitive contrast agents, we applied different labeling strategies and (1) electroporated electro-competent bacterial cells, (2) incubated electro-competent bacterial cells or (3) incubated unmodified bacterial cells with iron oxide particles of different size and coating. Labeling efficiency was assessed by confocal fluorescence microscopy using rhodamine-labeled particles and Syto 9 for co-registration (Figure 1). Syto 9 is a DNA marker and generally labels all bacteria in a population with a green fluorescence [32,33]. Our results showed that the pivotal step was to manipulate the structure of the cell wall by preparing electro-competent cells (Figure 1A-C). Electroporation of electro-competent *S. aureus* bacteria in the presence of 5-nm citrate-coated IONPs, according to BIO-RAD standard protocols [34,35], produced iron-labeled bacteria (Figure 1A). However, the procedure resulted in 47 ± 22% cell death, visible as iron-labeled cell debris by electron microscopy (Figure 1E) and precluded the method as labeling strategy for imaging experiments. On the other hand, when unmodified cells were simply incubated with IONPs, no fluorescence signal was detectable (Figure 1B). In contrast, after simple incubation of competent *S. aureus* bacteria with iron particles the rhodamine fluorescence signal was observable at the cell surface (Figure 1C). Compared to untreated cells the iron-labeled cell population showed the same natural cell death. Electron microscopy confirmed that clusters of IONPs were bound to the proteoglycane matrix that surrounds the cell (Figure 1F). An iron concentration of 1 μmol Fe per ml and 10^8^ bacteria was required to achieve sound labeling. By atomic spectrometry the iron load per cell was determined as 0.015 ± 0.002 pg Fe.

Larger particles or particles with other surface properties did not result in a homogeneous labeling of the cells, as shown by fluorescence microscopy (Figure 1D, Additional file 1). All studied 100-nm IONPs, independent from coating and surface charge, formed large particle aggregates which were attached to sporadic cells (see Additional file 1). When electro-competent *S. aureus* bacteria were incubated with 5-nm particles, only particles with citrate-coating led to a uniform iron labeling, while amine-coated particles labeled the cell suspension very inhomogeneously and resulted in scattered red fluorescent bacteria (Figure 1C, D). In contrast, for Gram-negative *E. coli* bacteria good labeling results were achieved both for citrate- and amine-coated IONPs leading to a labeling efficiency of 0.024 ± 0.003 pg/cell and 0.060 ± 0.004 pg/cell respectively (see Additional file 2).

It is known that electro-competent cells have a weakened cell wall with pores in the structure [36,37] and a much more homogeneous distribution of positively and negatively charged ions on the peptidoglycan matrix, providing an enlarged surface for interactions between charged iron particles and the cell wall. In Gram-positive *S. aureus* bacteria the cell wall mainly consists of a thick peptidoglycan layer giving rise to a positive net charge which may explain the high labeling efficiency when citrate-coated iron particles were used. In contrast, in Gram-negative *E. coli* bacteria the negatively charged lipopolysaccharide outer membrane leaf [38], which envelopes the cell, may explain the good labeling results for amine-coated IONPs.

### *In vitro* assays

**Figure 2:**
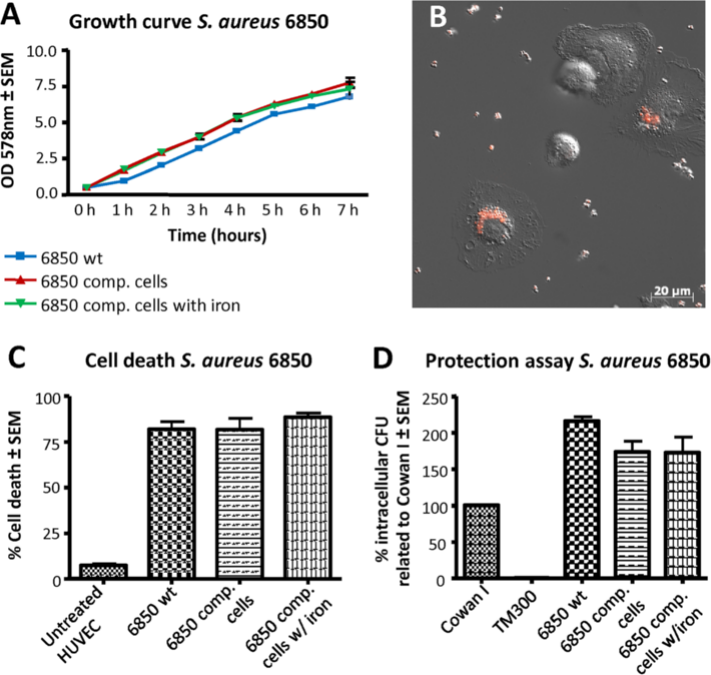
***In vitro *assays of iron-labeled *S. aureus *bacteria on HUVEC cells and macrophages.** Iron-labeled and non-labeled *S. aureus* bacteria (strain 6850) were compared to each other with regards to growth behavior, their cytotoxicity and invasiveness in HUVEC cells as well as their phagocytosis by macrophages. (**A**) In growth curves of *S. aureus* wild type cells (6850 wt) and electro-competent cells with (6850 comp cells w /iron) and without (6850 comp cell) iron particles, no significant differences were observed for the three cell cultures. (**B**) The fluorescence microscopic image shows how iron-labeled bacteria are phagocytized by macrophages. (**C**) In the cytotoxicity assay, cell death of HUVEC cells was compared and quantified upon treatment with *S. aureus* wild type cells, electro-competent and iron labeled bacteria. (**D**) In the invasion assay the invasiveness of the laboratory strain Cowan I was set as 100%, the non-invasive laboratory strain TM300 was used as a negative control. Neither growth behavior, cytotoxicity, invasiveness nor phagocytosis by macrophages was affected by the iron label.

To exclude a possible influence of the labeling procedure on bacterial cell characteristics we investigated growth behavior as well as cytotoxicity and invasion of iron-labeled bacteria. Growth curves did not differ significantly between untreated cells, and competent cells either with or without iron incubation (Figure 2A). Furthermore, infection models in HUVECs did not reveal differences between iron-labeled and unlabeled bacteria regarding cytotoxicity (Figure 2C) and invasive capacity (Figure 2D).

Most iron oxide particles have the formula Fe_2_^3+^O_3_Fe^2+^O, embedding the bi- and trivalent iron ions in a crystalline structure, which is chemically protected by molecular coatings. In our labeling procedure the iron particles are attached to the cell wall instead of being incorporated by the bacteria. This approach avoids bactericidal iron ions entering the bacteria, which may otherwise lead to toxic effects as described for iron complexes [39].

One of the most important types of immune cells recognizing invading bacteria *in vivo*, are macrophages. Therefore, we investigated in *in vitro* experiments whether bacteria carrying iron particles can still induce phagocytotic responses of macrophages. After adding iron-labeled bacteria to a suspension of murine macrophages, live microscopy showed that macrophages immediately started phagocytizing the labeled bacteria (Figure 2B, Additional file 3). These data show that iron labeling does not abrogate phagocytosis.

### Binding stability

**Figure 3:**
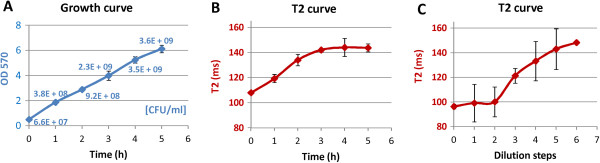
**Iron dilution during cell growth determined by MRI.** (**A**) Cell density and (**B**) transverse relaxation time (T2) of iron-labeled *S. aureus* samples at different time points of the growth curve. Cell cultures were labeled with 5-nm citrate coated IONPs (1 μmol Fe/ml for 10^8^ cells). For MRI measurements bacterial samples at each time point were diluted to an initial cell concentration of OD 0.5 and were embedded in 500 μl 0.5% agarose. (**C**) T2 measurements of agarose embedded cell cultures labeled with different iron concentrations in a 1:2 dilution row: 1 μmol Fe/ml, 0.5 μmol Fe/ml, 0.25 μmol Fe/ml, 0.125 μmol Fe/ml, 0.063 μmol Fe/ml, 0.031 μmol Fe/ml, 0.016 μmol Fe/ml. 1:2 dilution steps of the initial iron concentration imitate the iron dilution caused by cell division cycles. Note that dilution step 5 in (**C**) corresponds with measurement at 3 h in (**B**).

For the use of MRI to detect and track bacteria in animal models, it is of the utmost importance that the label is conserved over time and bacteria remain detectable after several growth cycles. Electron microscopy showed that IONPs remained bound to the surface after cell division and were passed on to the daughter cells (Figure 1F). Thus, each cell division step continuously led to a dilution of IONPs. To determine how fast the iron on the cell wall is diluted during cell growth and to estimate the minimum iron concentration that is detectable by MRI, growth curves of labeled bacteria were measured using an initial inoculum of an OD (optical density) value of 0.5. Every hour OD values and CFU (colony forming units) counts were determined and corresponding T1 (longitudinal relaxation time) and T2 (transverse relaxation time) values were measured at 9.4 T from a sample diluted to the initial cell concentration of 0.5 OD. Prior to MRI measurements the corresponding cell samples were separated from the medium and were embedded in 500 μl of 0.5% agarose. While T1 did not change significantly over the time course, T2 was significantly decreased at the beginning of the growth curve and increased steadily until a stationary value was reached after 3 h (Figure 3A, B, Additional file 4). According to the OD values and CFU counts, the cell density increased 35-fold during the first 3 h which resulted in a cell concentration of 2.3 × 10^9^ cells/ml. This observation confirmed that the binding of IONPs to the cell surface is stable and can be detected at least over five cycles of cell division. To assess the sensitivity and reliability of detection, we reversed the culture experiment and measured T2 of bacteria cultures labeled with a serial two-fold dilution of IONPs (Figure 3C). The corresponding T2 values indicated that bacteria were labeled to saturation for the initial dilution steps. From the third dilution on, T2 increased continuously with decreasing amount of iron. The T2 relaxation times measured after different cell division cycles (Figure 3B) correlated well with those obtained from cells labeled with different iron dilutions. T2 relaxation times of 121 ms and 142 ms, which were measured with a 1:8 and 1:32 iron dilution were also obtained from the growth curve at 1.2 h and 3 h, perfectly corresponding to three and five cell divisions.

### *In vivo* mouse models

**Figure 4:**
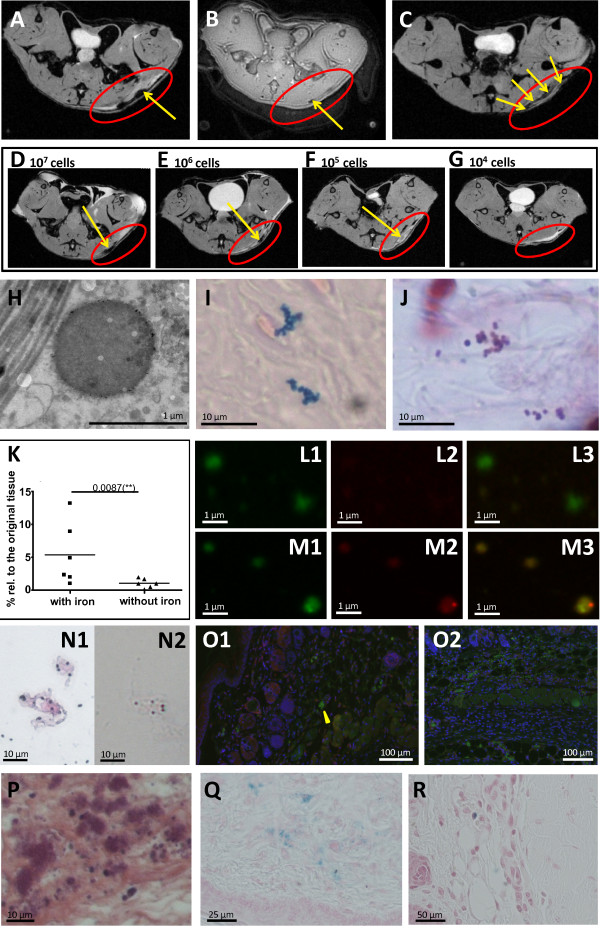
**MR images of a subcutaneous infection induced by iron-labeled bacteria investigated 24 h post infection.** Axial T2*-weighted (**A**) and UTE (**B**) images of the mouse flank, revealing an ulcerous abscess (red circle) with bacterial colonization (yellow arrows). (**C**) Axial T2*-weighted MRI of the subcutaneous infection with unlabeled bacteria, following intravenous co-injection of 5-nm IONP 2 h and 5 h post infection. (**D**-**G**) Axial T2*-weighted MRI of the mouse flank revealing an ulcerous abscess (red circle) with bacterial colonization (yellow arrows). The local skin infection was induced by different numbers of iron-labeled bacteria (**D**) 10^7^ CFU, (**E**) 10^6^ CFU, (**F**) 10^5^ CFU, (**G**) 10^4^ CFU. Bacteria were observed as hypointensities (yellow arrow) when iron-labeled *S. aureus* at a number ≥10^5^ CFU were applied. (**H**) Electron microscopy, (**I**) Prussian blue staining and (**J**) Gram staining of tissue sections of the inflammatory lesion showing iron-labeled bacteria. (**K**) CFU counts after magnetic separation of homogenized tissue infected with either iron-labeled or non-labeled *S. aureus* bacteria. (**L** and **M**) Fluorescence microscopy of homogenized infected tissue visualized in the green (1), red (2) and merged (3) fluorescence channel. Either unlabeled (**L**) *S. aureus* bacteria or bacteria labeled with rhodamine-coated IONPs (red, **M**) were used to induce the infection. For co-registration all bacteria were labeled with Syto 9 (green). (**N**) Gram (N1) and Prussian blue (N2) staining of homogenized tissue infected with iron-labeled bacteria. (**O**) Immunohistology of *S. aureus* infected skin tissue showing intracellular (yellow arrowhead, O1) and extracellular (O2) bacteria. Bacteria are stained green, macrophages red and nuclei blue. (**P** and **Q**) Hematoxylin/Eosin (**P**) and Prussian blue (**Q**) staining of tissue section of a lesion after subcutaneous infection with unlabeled *S. aureus*, followed by intravenous co-injection of 5-nm IONP 2 h and 5 h post infection. (**R**) Prussian blue staining of inflamed tissue with macrophage invasion, after infection with iron-labeled *S. aureus* bacteria.

For mammalian cells it has been shown previously that very low cell numbers, down to a single cell, can be detected and tracked by MRI [40]. Due to the very small size of bacteria (0.5 to 1 μm diameter) and the different labeling procedure, the feasibility of detection as well as its limits had to be assessed. To this end a local skin infection was induced by subcutaneous administration of either IONP-labeled or unlabeled *S. aureus* in the left flank of the mice. Formation of an ulcer 24 h post infection was obvious in MR images, showing the resulting edema as extended hyperintensity (Figure 4A). *S. aureus* colonies were identified as clearly circumscribed hypointensities in T2*-weighted images (Figure 4A). While T2*-weighting enhances the size of the actual colony, UTE (ultra-short echo time) MRI was able to visualize the extent of the colony more exactly (Figure 4B). However, for lower numbers of bacteria the blooming effect of T2*-weighting was required to detect the bacteria. The MRI detection limit of bacteria was assessed by infections with different numbers of labeled bacteria (10^4^, 10^5^, 10^6^, 10^7^ CFUs) (Figure 4D-G). A circumscribed hypointensity within the zone of inflammation was still reliably detectable in T2*-weighted images for 10^5^ bacteria CFUs, the same order of magnitude as found for BLI and fluorescence imaging [13]. Electron microscopy and Prussian blue staining of tissue sections 24 h post infection confirmed that IONPs were still bound to the cell surface giving rise to the observed hypointensities. Iron staining reproduced clusters of coccal-shaped bacteria observed in Gram stained sections of the inflamed tissue (Figure 4H-J). One of the big challenges in molecular MRI is to quantify the negative contrast caused by iron particles. Most studies have therefore focused on the qualitative assessment of hyper- and hypointense tissue contrast. Only few approaches have so far quantitatively tracked iron labeled cells, using, for example, shifted spin echo ultrashort T2* [41] or quantitative susceptibility mapping [42]. In our study, to quantify the amount of iron particles which is still bound on living bacteria 24 h post infection, we determined the iron concentration of bacterial colonies recovered from infected tissue on agar plates. From 10^7^ cells, which were injected subcutaneously to induce the infection, 3.92 × 10^6^ ± 3.18 × 10^6^ cells/ml per g tissue were recovered after 24 h, providing an iron content of 7.9 μg Fe per liter which corresponds to an iron concentration of 0.0020 ± 0.0016 pg Fe per cell. Compared to the initial labeling efficiency of 0.015 ± 0.002 pg Fe, the iron concentration per cell is approximately diluted 10-fold. By magnetic separation, which acts on bacteria that carry the iron label, 5.4 ± 4.8% of the recovered bacteria population could be separated from homogenized tissue (Figure 4K). However, the extracted bacterial number strongly depended on the iron distribution on single bacteria and bacterial clusters and, thus, represents only a rough estimation with pronounced variation between different tissue samples. After staining the recovered cell suspensions with Syto 9, the red fluorescence of the iron particles was co-localized with the detected bacteria (Figure 4M1-M3), confirming that the iron was still bound to the bacteria. The presence of intact bacteria in the tissue homogenate was confirmed by Gram and iron staining (Figure 4N1, N2). To assess the fraction of phagocytosed bacteria, 24 h post infection, tissue sections were stained with macrophage and *S. aureus*-specific antibodies. Intra- and extracellular bacteria were counted and averaged over 20 sections. Only a small fraction of 10% of the total bacterial number was observed intracellularly (yellow arrowhead, Figure 4O1, O2).

The particular strength of MRI for bacterial detection lies in its independence from metabolic activity and oxygenic environment, making it possible to also follow intracellular metabolically inactive *S. aureus*, such as the clinically highly relevant small colony variants [43,44]. For many bacterial pathogens and particularly for *S. aureus,* the intracellular persistence is increasingly recognized as an important infection and immune evasion strategy that causes chronic and therapy-refractory infections [45]. It has been demonstrated that *S. aureus* can invade and survive in different cell types, including professional phagocytes [46]. The use of iron labeled bacteria allowed us to track the surviving bacterial population in macrophages. Iron of digested bacteria is also distributed by immune cells over the time course, but these give rise to a different MRI pattern. Macrophage labeling by i.v. injection of IONPs 2 h and 5 h post infection resulted in a circumferential dark pattern at the border of the inflammation (Figure 4C) without clear hypointensities that indicated bacterial colonies. Histologically, large, not circumscribed areas of distributed iron were detected in zones of intense macrophage invasion (Figure 4P, Q). By contrast, after infection with iron labeled bacteria only sparely distributed single macrophages were labeled with Prussian blue (Figure 4R).

**Figure 5:**
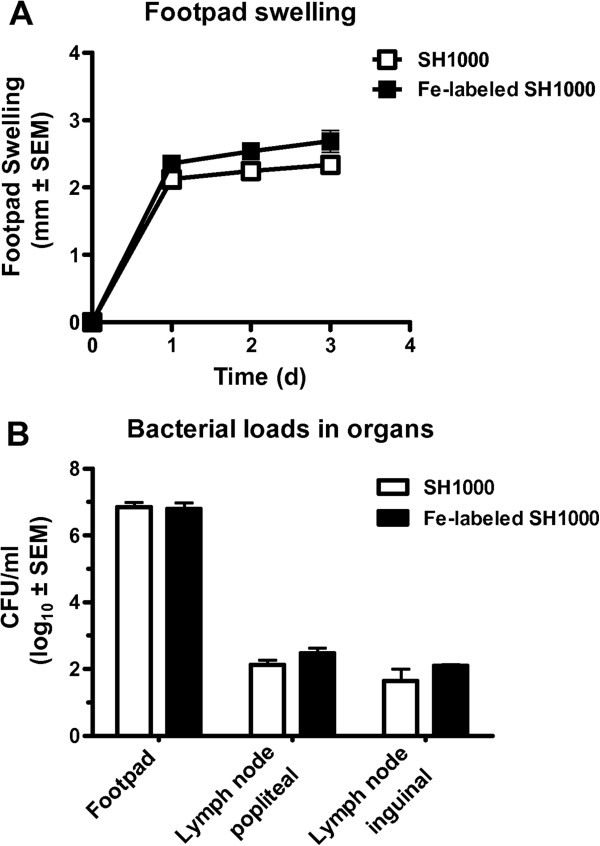
**Footpad infection model induced by unlabeled and iron-labeled *S. aureus *bacteria.** (**A**) Time course of footpad swelling and (**B**) bacterial load in infected footpads and draining popliteal and inguinal lymph nodes at three days after infection.

**Figure 6:**
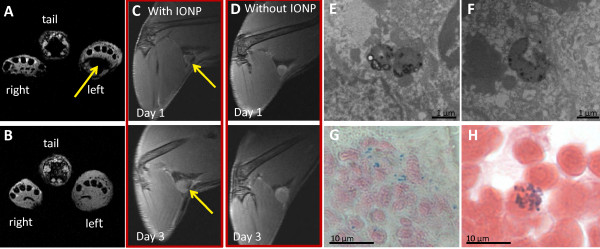
**MR images of a footpad infection induced by iron-labeled *S. aureus *bacteria.** Axial T2*-weighted MRI of the inflamed left footpad induced with (**A**) and without (**B**) iron-labeled bacteria, 24 h post infection. Bacteria were observed as hypointensities in the footpad (yellow arrow) when iron-labeled *S. aureus* were applied. (**C**, **D**) T2-weighted MRI of inflamed lymph nodes, Day 1 and Day 3 post infection. Bacteria were observed as hypointensities in the lymph nodes (yellow arrow) when iron-labeled *S. aureus* were applied. Electron microscopy (**E**, **F**), Prussian blue staining (**G**) and Gram staining (**H**) of the lymph node, 24 h post infection, showing iron-labeled bacteria.

The feasibility to track *S. aureus in vivo* was demonstrated in a footpad infection model which regularly involves draining lymph nodes [47]. IONP-labeled *S. aureus* (2 × 10^7^ CFU) were injected into the footpad, causing an inflammatory reaction (Figure 5). Similar to the local infection model, labeled bacteria were detected at the site of injection (Figure 6A, B). Beyond that, distant from the injection site, 1 d and 3 d after infection labeled bacteria were observed as hypointensities in the popliteal lymph node of the infected leg (Figure 6C). In contrast, lymph nodes of the infected leg after injection of unlabeled bacteria showed swelling as a sign of a systemic infection, but no typical hypointensities (Figure 6D). Popliteal lymph nodes of the uninfected leg did not show signs of inflammation and lymph nodes of mice being injected with iron particles only, did not show hypointensities (see Additional file 5). Electron microscopy, Gram and Prussian blue staining of tissue sections confirmed the presence of iron-labeled bacteria in the contrasted lymph nodes (Figure 6E-H). These data show that disseminating bacteria are detectable by MRI. Even though BLI is a well-established method of studying bacterial infections in animal models and is superior to MRI with regards to ease of use, cost and scan time, it is strictly limited to preclinical studies. MRI on the other hand is directly amenable to study human subjects. Our MRI approach to track bacteria *in vivo*, raises hope that this modality can be transferred into the clinics in the future, when pathogen-specific contrast agents have become available.

## Conclusions

In conclusion, we have established a novel modality for *in vivo* detection and tracking of bacteria, which is complementary to BLI. With the application of pre-labeled bacteria, the method is limited to use in animal models, as is BLI. The great advantage of MRI is the simultaneous imaging of morphology, organ compartments and immune response together with the data on bacterial tracking. Additionally, MRI detection is also applicable to metabolically less active as well as anaerobic bacteria and not subject to limited penetration depth. Therefore, we are confident that this modality greatly enhances the diagnostic and analytical options in fundamental and translational research on bacterial infections.

## Methods

### Bacteria and growth conditions

#### Bacterial strains

Experiments were performed with the two *S. aureus* bacterial strains, 6850 (ATCC 53657) [48] and SH1000 [49] which is a derivate of strain 8325–4 with restored rsbUþ-gene. As a Gram-negative strain *E. coli* K12 was used.

#### Cultivation

Bacteria were cultured in Mueller-Hinton (MH, *S. aureus*) or Luria Bertani (LB, *E. coli*) medium overnight at 37°C with shaking at 160 rpm and were subsequently used to prepare electro-competent cells or to perform *in vitro* assays.

#### Bacterial concentration

The cell concentration was determined by measuring the OD578 (optical density at 578 nm) spectroscopically and by counting the colony forming units (CFU). A bacterial sample was serially diluted and a known volume was plated on blood agar. The bacterial concentration was obtained by counting the number of colonies that had formed.

#### Electro-competent bacteria

Electro-competent *S. aureus* and *E. coli* bacteria were made according to the manufacturer’s protocol suggested for each microorganism [35] and were frozen in 10% glycerol in a concentration of 10^9^ to 10^10^ cells/ml.

### Labeling bacteria

To label bacteria, either electro-competent or naïve cells were used. A total of 50 μl of the cell suspension was incubated with 2 μl of iron oxide particles in 950 μl MH or LB medium for 1 h at 37°C and 160 rpm. The following IONPs were used: VSOP-rhodamine 5 nm and VSOP-link-rhodamine 5 nm (Very Small Iron Oxide Particles, both Ferropharm AG, Teltow, Germany), screenMAG 100 nm (diethylamine ethyl, phosphatidyl-cholin and poly(maleic acid-co-olefin), polysaccharide, all Chemicell, Berlin, Germany). VSOP particles consist of a 5 nm maghemit core and hold an iron concentration of approximately 2.2 × 10^-19^ g/per particle. Prior to use, cell suspensions were centrifuged at 5,000 rpm for 10 minutes to remove culture media as well as excessive IONPs and pellets were washed once with phosphate-buffered saline (PBS) and were resuspended in PBS. Labeling efficiency was visualized by fluorescence microscopy and bacteria were additionally labeled with Syto 9 (Invitrogen, Darmstadt, Germany) for co-registration according to molecular probes standard protocols.

### Lysostaphin protection assay

The invasiveness of *S. aureus* with or without iron labeling in endothelial cells was performed by lysostaphin protection assay. Two days prior to the assay 1 ml HUVEC (2 × 10^5^ cells/ml) was plated in 12-well plates in M199 medium (PAA Laboratories GmbH, Cölbe, Germany) and was stimulated with live bacteria. Bacteria from frozen stock suspensions were washed once with PBS and adjusted to a cell concentration of 5 × 10^8^ cells/ml in MH medium and were incubated for 1 h at 37°C and 160 rpm. When the assay was performed with iron-labeled bacteria, freshly prepared cultures according to the labeling protocol were used directly. Bacteria were harvested, washed once and resuspended in PBS. A total of 100 μl of the bacterial suspensions were added to each well. After an invasion period of 3 h, HUVEC were washed with PBS, the culture medium was replaced by M199 plus 10% FCS (fetal calf serum) and the HUVEC were incubated with lysostaphin (20 μg/ml) for 30 minutes at 37°C in order to remove extracellular staphylococci. The cells were washed with PBS and intracellular bacteria were released by incubation in 1 ml of H_2_O for 30 minutes at 37°C. Samples were diluted in PBS and plated on blood agar plates to determine the number of recovered CFUs. The assay was performed with a positive (Cowan I) and a negative control (TM300). The invasiveness of the laboratory strain Cowan I was set as 100%.

### Cell death assays

Analysis of cell death was performed by measuring the proportion of hypodiploid nuclei as described in Haslinger-Löffler *et al*. [50]. Briefly, HUVEC plated in 12-well plates were incubated for 3 h with 20 μl of 5 × 10^8^ CFU/ml *S. aureus* bacteria per well. Incubation with lysostaphin for 30 minutes followed. After the removal of extracellular staphylococci, HUVEC were washed with PBS and full medium was added. After 20 h the cell death was determined and cells were stained with the DNA-dye propidium iodide and analyzed with a FACS Calibur flow cytometer (Becton Dickinson, Heidelberg, Germany).

### Growth curve assay

In a 500 ml flask, 50 ml of bacterial staphylococcal cell suspension with an initial cell concentration of OD 0.5 was incubated for 7 h at 37°C and 160 rpm in MH medium. Every hour a sample was taken to measure OD and CFU counts/ml.

### Bacteria recovered from infected tissue

Approximately 0.1 g infected skin tissue was collected and homogenized in 2 ml PBS. An aliquot of 100 μl was plated on blood agar over night and the CFUs harvested and suspended in 1 ml PBS. The iron concentration of the cell suspension was subsequently measured by atomic spectrometry. To determine the iron concentration per cell the measured iron content was corrected by an iron baseline. Since iron is an important nutrient in agar plates, it is taken up during cell growth. As a baseline a defined number of *S. aureus* bacteria were cultured on blood agar plates, harvested, suspended in 1 ml PBS and measured by atomic spectrometry.

### Magnetic separation

The number of iron labeled bacteria in infected tissue was determined with the aid of magnetic separation. For this purpose, 1 ml tissue sample was placed in a magnetic field for five minutes, washed twice with PBS, diluted with 1 ml PBS and plated for CFU count. CFU counts of iron-labeled and unlabeled bacteria were analyzed by a Mann–Whitney-Test.

### Fluorescence microscopy

Labeled bacteria suspensions were added with 0.1% agarose and sealed by a second coverslip. Fluorescence images were obtained with a commercial 4Pi microscope (TCS 4Pi microscope type A, Leica Microsystems GmbH, Wetzlar, Germany) employing oil immersion lenses (×100, numerical aperture 1.46). The TCS 4Pi is a confocal laser scanning microscope of type TCS SP2 incorporating single as well as two-photon excitation, photon-counting by avalanche photodiodes, and a 4Pi attachment. Because of these features the microscope could be employed in the confocal mode with upright or inverted beam path and single- or two-photon excitation, or as a two-photon excitation 4Pi microscope. The images were taken in the confocal mode of the microscope. Due to the spherical shape of the *S. aureus* bacteria the 4Pi mode brings no significant gain in resolution. In a 4Pi image, every pixel has attached an interference function in z direction. If we take a picture of a sphere, these functions strongly overlap and cause blurring. Single-photon excitation wavelengths of 476 and 561 nm was used, yielding best case resolutions of 166 nm and 196 nm, respectively, in both x and y direction. The beam expander was set to 6. Fluorescence originating from the sample was passed through a filter cube (short-pass 700 nm, beam splitter 560 nm, band-pass 500 to 550 nm, and band-pass 607 to 683 nm), and its intensity was measured by photon-counting avalanche photodiodes (PerkinElmer, Waltham, MA, USA). The detection pinhole was set to 1 Airy unit. Images of the cells were recorded with a pixel size between 12 × 12 nm^2^ and 42 × 42 nm^2^. Raw images were linearly brightened, rescaled and linearly filtered by a subresolution mask using the image processing program ImageJ (Wayne Rasband, US National Institutes of Health, [51]).

### Live cell microscopy

Bone marrow-derived macrophages (BMDMs) were differentiated *in vitro* from bone marrow as previously described [52]. BMDMs were transferred to fibronectin-coated LabTek chamber slides and incubated for at least 12 h to achieve adherence. After that, live bacteria were added (MOI 50) and life cell imaging was performed using an inverted fluorescence microscope (Zeizz Axio Observer Z1; Carl Zeiss MicroImaging GmbH, Jena, Germany; 40× objective magnification).

### Histology

For histopathology, cryo and paraffin sections of lymph node and skin tissue were prepared and Gram-positive bacteria were stained with crystal violet/iodine and Prussian blue according to standard protocols. Macrophages were stained with hematoxylin and eosin. Bacteria and macrophages were visualized using a light microscope (Zeiss Axioskop, Goettingen, Germany; 100×, 40×, 20× 10× objective magnification).

### Immunohistology

For immunohistology, paraffin embedded sections of infected skin tissue (5 μm) were prepared on slides and blocked with 2% BSA. Macrophages and *S. aureus* bacteria were stained according to standard protocols using an appropriate dilution of a monoclonal rat anti-mouse F4/80 antibody (clone BM8, purchased from Biolegend, Fell, Germany) or a polyclonal rabbit anti-S. aureus IgG (Abcam, Cambridge, UK) followed by incubation with AlexaFluor 568-conjugated anti-rat and AlexaFluor 488-conjugated anti-rabbit secondary antibodies (both purchased from Invitrogen, Karlsruhe, Germany). Nuclei were counterstained using 0.1 μg/ml 4′,6-diamidino-2-phenylindole (DAPI). Macrophages and *S. aureus* bacteria were visualized by fluorescence microscopy using an Olympus BX63 microscope and the CellSens Software (Olympus, Münster, Germany). Immunofluorescence staining is depicted in an original magnification of 100×.

### Electron microscopy

Mice were sacrificed and intravenously perfused with modified Karnovsky’s fixative consisting of 2.5% glutaraldehyde, 2% paraformaldehyde in PBS. After fixation of the infected organ or tissue, samples were dissected and placed in fresh fixative and kept overnight at 4°C. Subsequently, tissue was washed three times with 0.1 M cacodylat buffer for 5 to 10 minutes, cut into pieces ≤1 mm and post-fixed with 2% osmium tetroxide at room temperature for 1 h. The samples were extensively washed with 0.1 M cacodylat buffer and stained with 1.5% uranyl acetate for 1 h during dehydration in an ethanol series. In the last dehydration step after pure ethanol pure acetone was used. Tissue samples were then embedded in Epon 812. Ultrathin sections (<60 nm) were collected on Formvar coated copper grids and viewed with a Phillips CM 10 or Zeiss Libra 120 (Zeiss, Oberkochen, Germany) electron microscope.

Iron-labeled bacteria samples were embedded in 1% agarose and then fixed for more than 24 h using the same fixative as for the animal tissues. Subsequent preparation steps were performed as described above.

In case of iron enhancement so called Perl’s reaction was used in between the primary fixation of the samples with aldehyde mixture and post-fixation with osmium. The procedure was performed as described in Parmley *et al*. [53].

### Atomic spectrometry

Gallium standard solution (c = 1.000 mg/l) in the highest quality available was purchased from Fluka Chemie GmbH (Buchs, Switzerland). Total reflection X-ray fluorescence analysis (TXRF) was carried out on a S2-PICOFOX instrument (Bruker AXS, Berlin, Germany) with an air-cooled molybdenum anode for X-ray generation. The excitation settings were 50 kV and 750 mA and quartz glass disks were used as sample carriers. As internal standard, gallium with a concentration of 10 mg/l was applied. Aliquots of the samples were mixed with the same volume of the 10 mg/l gallium standard solution, placed on the sample carriers and evaporated to dryness. The analysis was performed by signal integration over 1.000 seconds. For the determination, the peaks of iron (Kα1 = 6.3921 keV) and gallium (Kα1 = 9.251 keV), as internal standard, were used. Quantification was performed by the Bruker Spectra software (version 6.1.5.0) and based on the known concentration of the internal gallium standard.

### Magnetic resonance imaging (MRI)

Images were acquired at 9.4 T on a Bruker BioSpec 94/20 (Ettlingen, Germany).

#### In vivo measurements

The subcutaneous lesions of infected mice were measured with a 1 T/m gradient system and a 35 mm volume coil using FLASH and UTE sequences with the following parameters (FLASH: TR: 1,500 ms, TE: 6 ms, FA: 30°, FOV: 3.20 × 3.20 cm, MTX: 256 × 256; UTE: TR: 100 ms, TE: 314 μs, FA: 20 deg, FOV: 3.20 × 3.20 cm, MTX: 256 × 256). For lymph node imaging a 0.7 T/m gradient system and a surface coil was used and images were acquired by a TurboRARE sequence (TR: 4.5 ms, TE: 9.3 μs, FOV: 4.50 × 2.50 cm, MTX: 512 × 312).

#### In vitro measurements

A total of 500 μl of iron-labeled *S. aureus* suspensions in a concentration of OD 0.5 were washed with PBS and suspended in 500 μl of a 0.5% agarose solution. T1 and T2 measurements were performed using a 0.7 T/m gradient system and a 72 mm volume coil by using the following pulse sequences: RAREVTR: TE: 10 ms, 30 ms, 50 ms, 70 ms, 90 ms, 110 ms, 130 ms; TR: 5,500 ms, 3,000 ms, 1,500 ms, 800 ms, 400 ms, 200 ms; average: 1; slice thickness: 1.5 mm; MTX: 128 × 128; RARE factor: 2; FOV: 23 mm × 40 mm. MSME: TE: 11 ms, 22 ms, 33 ms, 44 ms, 55 ms, 66 ms, 77 ms, 88 ms, 99 ms, 110 ms, 121 ms, 132 ms, 143 ms, 154 ms, 165 ms, 176 ms, 187 ms, 198 ms, 209 ms, 220 ms, 231 ms, 242 ms, 253 ms, 264 ms, 275 ms, 286 ms, 297 ms, 308 ms, 319 ms, 330 ms; TR: 2,500 ms; average: 1; slice thickness: 1.5 mm; MTX: 256 × 256; FOV: 23 mm × 40 mm.

### Mice

Female C57BL/6 wild-type mice 8- to 12-weeks old were purchased from Harlan-Winkelmann (Borchen, Germany) and kept one week at the animal facility of the Translational Research Imaging Center, Muenster, Germany, before the beginning of the experiments. All animal studies were performed with the approval of the State Review Board of North Rhine-Westphalia, Germany (TVA-Nr. 87–51.04.2001.A003, TVA-Nr. 8.87-50.10.36.08.194).

### Animal models

#### Subcutaneous infection

The infection was induced in the left flank of C57BL/6 mice by subcutaneous injection of 100 μl of either iron-labeled or unlabeled *S. aureus* suspensions (1 × 10^8^ CFU/ml in PBS). N = 6 mice were used to assess the fraction of iron-labeled bacteria 24 h post infection.

#### Systemic lymph node infection

Mice were inoculated subcutaneously with 2 × 10^7^ CFU of iron-labeled or unlabeled *S. aureus* bacteria, by injecting 50 μl of the cell suspensions (4 × 10^8^ cells/ml in PBS) into the left hind footpad as described previously by Nippe *et al*. [47]. Control animals injected with the same amount of PBS or PBS-based iron suspension showed no significant swelling reaction. For each group of infected mice four or five mice were used. Footpad swelling was measured daily with a micrometric caliper, and specific footpad swelling was assessed by subtracting the diameter of injected (left) from non-injected (right) footpad. Lymph node swelling and bacterial distribution was observed by MRI. Mice were sacrificed by CO_2_ asphyxiation after the MRI measurements and lymph nodes were either used for histology, electron microscopy or CFU counts. Colonies were counted after incubation of homogenized tissue overnight at 37°C.

#### Macrophage detection

Two hours and five hours post infection, mice were injected i.v. with 100 μl of 5-nm IONP (VSOP, Ferropharm AG, Teltow, Germany) suspensions (300 μmol/kg body weight).

## Abbreviations

BLI: Bioluminescence imaging; BMDM: Bone marrow-derived macrophages; CFU: Colony forming unit; CT: Computed tomography; FCS: Fetal Calf Serum; FLASH: Fast Low-Angle Shot; FOV: Field of view; HUVEC: Human Umbilical Vascular Endothelial Cell; IONP: Iron oxide nano particle; LB: Luria Bertani; MH: Mueller-Hinton; MTX: Matrix; MRI: Magnetic resonance imaging; MSME: Multi Slice Multi Echo; OD: Optical density; PBS: Phosphate buffered saline; PET: Positron emission tomography; RARE: Rapid Acquisition with Refocused Echoes; TXRF: Total reflection X-ray Fluorescence; T1: Longitudinal relaxation time; T2: Transverse relaxation time; TE: Echo time; TR: Repetition time; UTE: Ultra-short echo time.

## Competing interests

The authors declare that they have no competing interests.

## Authors’ contribution

VH coordinated the study, designed experiments, performed MRI and cell labeling experiments, collected and analyzed data and wrote the manuscript. LT performed cell labeling and cell culture experiments and analyzed data. JH performed and validated the fluorescence microscopy experiments. NN established and characterized the footpad infection model, acquired and analyzed data. KL performed immunohistology and analysis. NG and YT performed the electron microscopy. MH performed atomic spectrometry. JK, CS, UK, GP and BL analyzed data. CF developed the concept of the study, analyzed data and wrote part of the manuscript. All authors edited the manuscript. All authors read and approved the final manuscript.

## Supplementary Material

Additional file 1 **Fluorescence microscopy of labeled *S. aureus *bacteria.***S. aureus* labeled with Syto 9 (green) and rhodamine-coated iron oxide particles (red). (**A**) screenMAG 100 nm polysaccharide, (**B**) screenMAG 100 nm diethylamine ethyl, (**C**) screenMAG 100 nm phosphatidyl-cholin, (**D**) screenMAG 100 nm poly(maleic acid-co-olefin). Click here for file

Additional file 2 **Fluorescence microscopy of labeled *E. coli *bacteria.***E. coli* labeled with Syto 9 (green) and rhodamine-coated iron oxide particles (red) by using either (**A**) amine-coated 5-nm IONPs or (**B**) citrate-coated 5-nm IONPs. Click here for file

Additional file 3 **Murine macrophages phagocytizing iron-labeled bacteria.** The movie shows how iron-labeled *S. aureus* bacteria (red) are phagocytized by murine macrophages. Click here for file

Additional file 4 **Longitudinal and transverse relaxation times of iron-labeled *S. aureus *cultures, measured at different growth stages.** The measurements of the relaxivities showed that T1 did not change significantly over the time course, while T2 was significantly decreased at the beginning of the growth curve and increased steadily until a stationary value was reached after 3 h. Click here for file

Additional file 5 **MR images of the left footpad injected with IONPs.** (**A**) IONPs in the footpad were observed as hypointensities (yellow arrow) in axial T2*-weighted MRI. (**B**) T2-weighted images of the popliteal lymph node on Day 2 post injection did not show hypointensities as was observed for a footpad infection induced by iron-labeled bacteria. Click here for file
