# Supplementary figures and images for: Bacteria tracking by in vivo magnetic resonance imaging
Source: BMC Biol. 2013 May 28;11:63. doi: 10.1186/1741-7007-11-63 (PMC3686665; doi:10.1186/1741-7007-11-63)

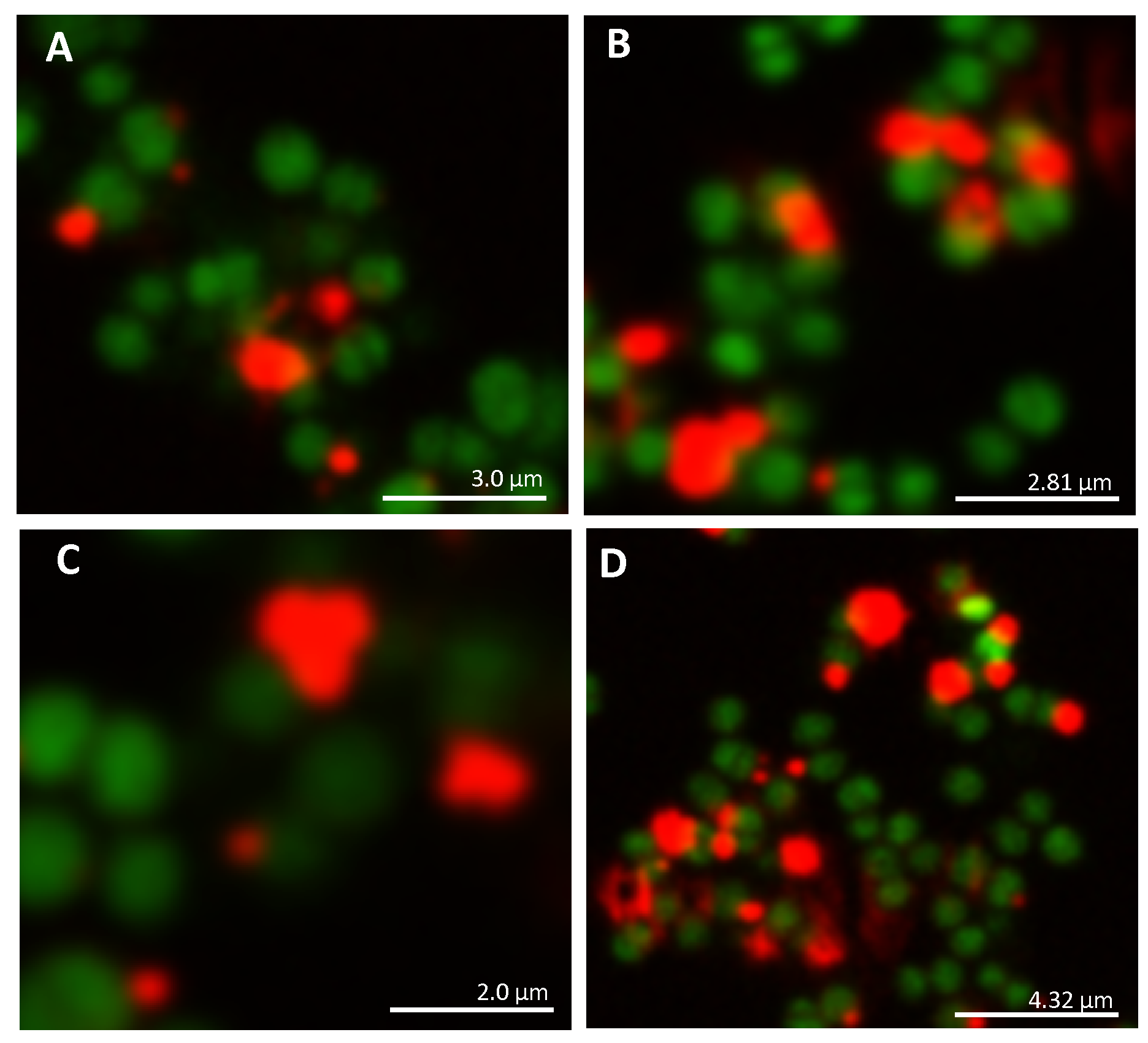

Supplement: Additional file 1 — Fluorescence microscopy of labeled S. aureus bacteria.S. aureus labeled with Syto 9 (green) and rhodamine-coated iron oxide particles (red). (A) screenMAG 100 nm polysaccharide, (B) screenMAG 100 nm diethylamine ethyl, (C) screenMAG 100 nm phosphatidyl-cholin, (D) screenMAG 100 nm poly(maleic acid-co-olefin). [file 1741-7007-11-63-S1.tiff]

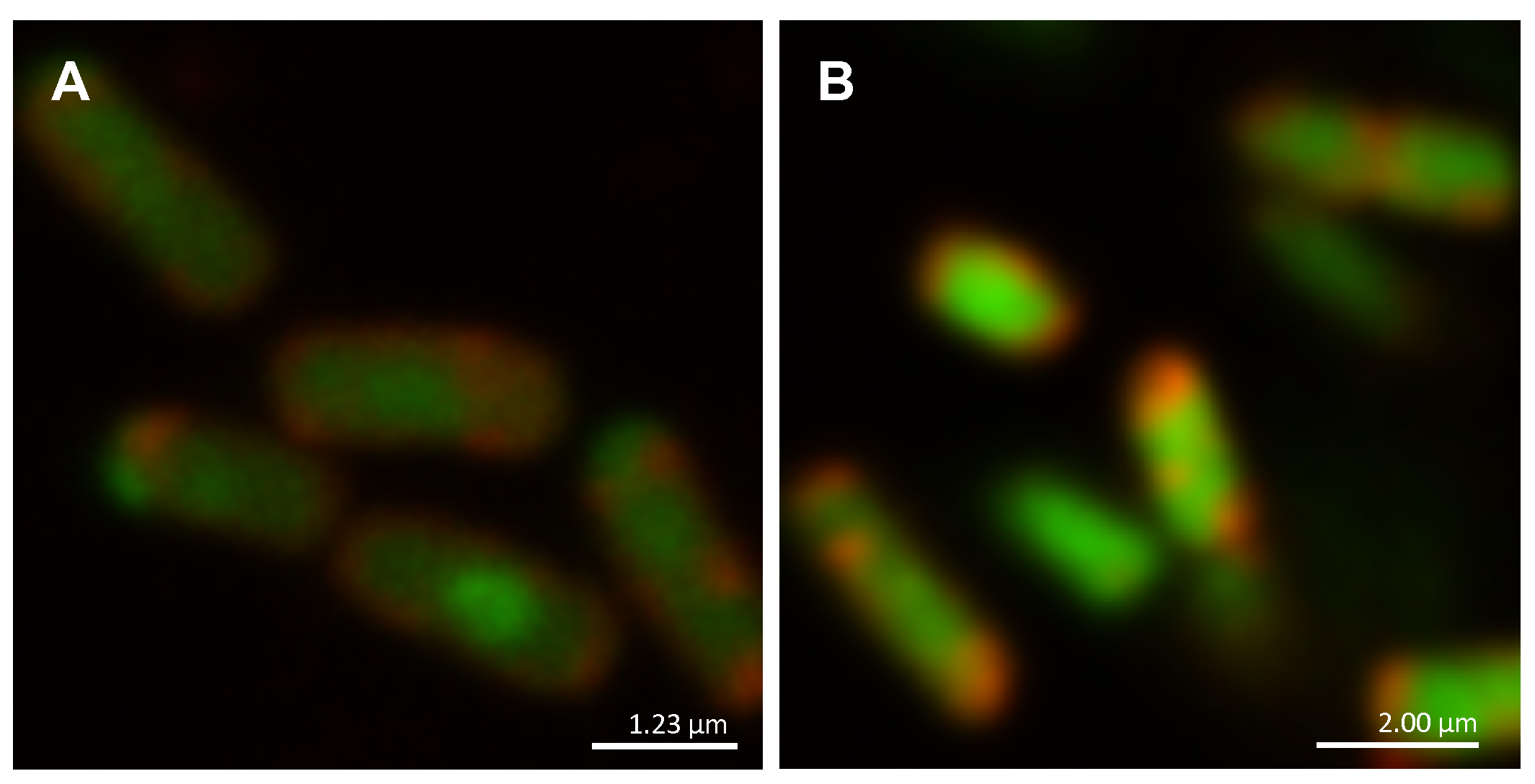

Supplement: Additional file 2 — Fluorescence microscopy of labeled E. coli bacteria.E. coli labeled with Syto 9 (green) and rhodamine-coated iron oxide particles (red) by using either (A) amine-coated 5-nm IONPs or (B) citrate-coated 5-nm IONPs. [file 1741-7007-11-63-S2.tiff]

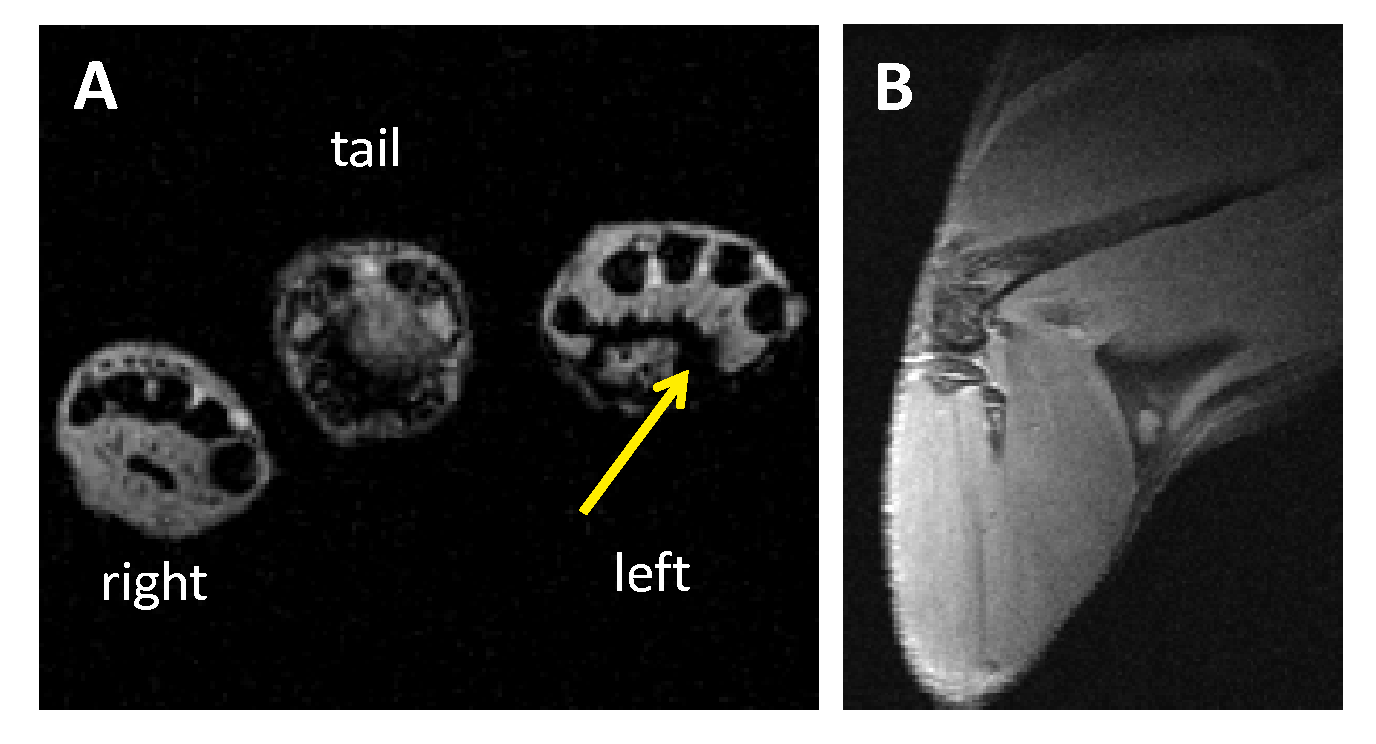

Supplement: Additional file 5 — MR images of the left footpad injected with IONPs. (A) IONPs in the footpad were observed as hypointensities (yellow arrow) in axial T2*-weighted MRI. (B) T2-weighted images of the popliteal lymph node on Day 2 post injection did not show hypointensities as was observed for a footpad infection induced by iron-labeled bacteria. [file 1741-7007-11-63-S5.tiff]
